# Monoklonale Antikörper zur antiinfektiven Therapie

**DOI:** 10.1007/s00103-020-03229-1

**Published:** 2020-10-09

**Authors:** Bettina Klug, Barbara Schnierle, Isabel Trebesch

**Affiliations:** 1 Paul-Ehrlich-Institut, Paul-Ehrlich-Straße 51–59, 63225 Langen, Deutschland; 2 Robert Koch-Institut, Berlin, Deutschland

**Keywords:** Immuntherapie, Anti-virale monoklonale Antikörper, Anti-bakterielle monoklonale Antikörper, Symptomatische Behandlung, SARS-CoV-2, Immunetherapy, Anti-viral monoclonal antibodies, Anti-bacterial monoclonal antibodie, Symptomatic treatment, SARS-CoV-2

## Abstract

Ein Jahrhundert lang wurde die Serumtherapie von Seren tierischen Ursprungs und Hyperimmunglobulinen dominiert. Obwohl seit Ende der Achtzigerjahre des letzten Jahrhunderts zahlreiche monoklonale Antikörper (MAB) insbesondere zur Behandlung von immunologischen und onkologischen Erkrankungen entwickelt wurden, sollte es noch 20 Jahre bis zur Zulassung des ersten antiinfektiven MAB in der Europäischen Union dauern. In den folgenden 2 Dekaden kamen nur 2 weitere antiinfektive MAB hinzu. Interessanterweise werden zurzeit zur Bekämpfung der COVID-19-Pandemie zahlreiche MAB, die insbesondere in immunologischer Indikation zugelassen sind, zur Behandlung der Folgen der SARS-CoV-2-Infektion, wie Pneumonie oder Hyperimmunreaktion, eingesetzt.

Im Folgenden werden die zugelassenen monoklonalen Antikörper zur Behandlung von Infektionskrankheiten vorgestellt. Darüber hinaus wird eine Übersicht über die aktuellen Entwicklungen, insbesondere bei der Therapie der SARS-CoV-2-Infektion, gegeben.

## Abstract (en)

Sera of animal origin and hyperimmunoglobulins have dominated serum therapy for a century. Although numerous monoclonal antibodies (MABs) have been developed since the end of the 1980s, particularly for the treatment of immunological and oncological diseases, it will take 20 years before the first anti-infective MAB is approved in the European Union. Interestingly, to combat the COVID-19 pandemic, numerous MABs, which are approved in particular for immunological indications, are currently being used to treat the consequences of SARS-CoV‑2 infection, such as pneumonia or hyperimmune reactions.

The approved monoclonal antibodies for the treatment of infectious diseases are presented here. In addition, an overview of the current developments, in particular in the treatment of SARS-CoV‑2 infection, is provided.

## Einleitung

Mit dem Aufsatz „Über das Zustandekommen der Diphtherieimmunität und der Tetanusimmunität bei Thieren“ [1] begründete Emil von Behring zusammen mit dem Japaner Shibasaburō Kitasato die moderne Serumtherapie. Vier Jahre nach Erscheinen des Aufsatzes konnten Kinder in größerem Umfang erfolgreich mit einem Diphtherieserum behandelt werden [2].

Der Einsatz von Antiseren tierischen Ursprungs, sowie später auch von Hyperimmunglobulinen, gewonnen aus Plasma von immunisierten oder genesenen Spendern, zur Behandlung oder Vorbeugung von Infektionskrankheiten war in der Zeit vor der Verfügbarkeit von Impfstoffen und Antibiotika weitverbreitet. Antitoxine und Hyperimmunglobuline werden auch heute noch vorzugsweise zur sekundären Prävention nach möglicher Infektion oder zur Therapie von durch Toxine hervorgerufenen Erkrankungen oder viralen Infektionen, wie u. a. Diphtherie, Botulismus, Tollwut, Tetanus, Hepatitis A und B oder Masern, eingesetzt [3].

Mit der Entwicklung der Hybridomtechnologie durch Milstein, Köhler und Jernes im Jahr 1975 wurde die Entwicklung von monoklonalen Antikörpern (MAB) und die Produktion in großen Mengen ermöglicht [4].

Die Zulassung des MAB Muromonab-CD3 [5] zur Prävention der akuten Transplantabstoßung nach Nierentransplantation [6] Ende der Achtzigerjahre des letzten Jahrhunderts war der Durchbruch der monoklonalen Antikörpertherapie, insbesondere in immunologischen oder onkologischen Indikationen. Es dauerte aber noch fast 2 Jahrzehnte bis zur Zulassung des ersten antiinfektiven monoklonalen Antikörpers in der Europäischen Union (EU). Im Jahr 1999 wurde Palivizumab zur Prävention der durch das Respiratory-Syncytial-Virus (RSV) hervorgerufenen schweren Erkrankungen der unteren Atemwege, die Krankenhausaufenthalte erforderlich machten, bei Kindern mit hohem Risiko für RSV-Erkrankungen zugelassen [7]. Wiederum knapp 20 Jahre später wurden von der Europäischen Arzneimittel-Agentur (EMA) noch 2 weitere monoklonale Antikörper zur Behandlung von Infektionskrankheiten zugelassen: Bezlotoxumab zur Prävention der Rekurrenz einer *Clostridium-difficile*-Infektion (CDI) bei Erwachsenen mit einem hohen Rekurrenzrisiko einer CDI [8] und Ibalizumab in Kombination mit anderen antiretroviralen Arzneimitteln zur Behandlung von Erwachsenen mit einer multiresistenten HIV-1-Infektion, bei denen kein anderes supprimierendes, antivirales Regime Wirksamkeit zeigt [9].

Im Gegensatz zu den monoklonalen Antikörpern zur Therapie von nichtübertragbaren Krankheiten ist das Interesse an der Entwicklung von monoklonalen Antikörpern zur Therapie und Prävention von Infektionskrankheiten gering. Mit dem Auftreten und der Verbreitung von neuen Infektionskrankheiten (Emerging Diseases) ist das Interesse an der Entwicklung von antiinfektiven monoklonalen Antikörpern gestiegen.

Hier stellen wir die zugelassenen monoklonalen Antikörper zur Behandlung von Infektionskrankheiten vor. Darüber hinaus geben wir eine Übersicht über die aktuelle Entwicklung von neuen MAB bei Infektionskrankheiten, insbesondere zur Therapie von SARS-CoV-2-Infektionen.

## Eigenschaften von monoklonalen Antikörpern

Die modernen biotechnologischen Herstellungsverfahren von MAB führen zu Produkten mit einer sehr hohen Reinheit und hohen Ausbeuten und die Toxizität moderner humaner und humanisierter monoklonaler Antikörper ist gering [10]. Die Herstellung von MAB erfordert Zellkulturen oder mikrobielle Expressionssysteme. Im Gegensatz zur Serumtherapie, deren Ausgangsmaterial von menschlichen oder tierischen Spendern gewonnen wird, ist bei der Herstellung von MAB die Wahrscheinlichkeit einer versehentlichen Übertragung von Infektionskrankheiten reduziert [11].

Im Prinzip können die MAB direkt gegen bakterielle oder virale Antigene oder Toxine gerichtet sein (externe Antigene) und dadurch den Eintritt in die Zelle blockieren oder das Toxin neutralisieren. Ferner kann der Antikörper auch gegen zelluläre Rezeptoren gerichtet sein und so den Eintritt in die Zelle unterbinden [11–13].

Aufgrund der hohen Spezifität der Antikörper bergen sie das Risiko der Entwicklung von Escape-Mutationen, die zu einem Wirkverlust führen können. Das Risiko der Resistenzentwicklung kann durch den Einsatz von Antikörpercocktails, die sich gegen verschiedene Epitope richten, minimiert werden [12, 14, 15].

In jüngster Zeit werden auch immunmodulatorische MAB eingesetzt, um die Folgen der Entzündungsreaktion einer Infektion einzudämmen [16].

## Antivirale monoklonale Antikörper

Antivirale MAB können entweder eine breite neutralisierende Aktivität gegen hochkonservierte virale Epitope zeigen oder aber sie sind gegen Zellrezeptoren/Co-Rezeptoren der Wirtszelle gerichtet, um den Eintritt des Virus in die Wirtszelle zu verhindern [10]. Ein weiterer Ansatz sind MAB, die gegen infizierte Wirtszellen gerichtet sind und eine antikörpervermittelte Zytotoxizität induzieren [17].

Bisher sind in der EU ein MAB zur Therapie bei multiresistenten HIV-Infektionen sowie ein MAB zur Vorbeugung von RSV-Infektionen bei Hochrisikokindern zugelassen worden (EMA/CHMP: Trogarzo-EPAR Summary for the Public [18]).

## Antikörpertherapien gegen das Respiratory-Syncytial-Virus (RSV)

Der humanisierte monoklonale Antikörper Palivizumab wurde zur Prävention schwerer Erkrankungen der unteren Atemwege entwickelt, die bei Kindern mit hohem Risiko durch das RSV verursacht werden. RSV ist die häufigste Ursache für Infektionen der unteren Atemwege bei Säuglingen. Palivizumab bindet an die Region „Antigene Stelle II“ (auch „Stelle A“ genannt) des RSV-F-Glykoproteins von RSV und verhindert so die Virusvermehrung und die Entstehung schwerer Erkrankungen [19].

In einer placebokontrollierten Studie zur Prophylaxe der RSV-Erkrankung bei 1502 Kindern mit erhöhtem Infektionsrisiko führte die 5‑monatliche Gabe von Palivizumab in 55 % der Fälle (*p* = <0,001) zu einer Reduzierung der RSV-bedingten Krankenhausaufnahmen. Die Schwere der RSV-Erkrankung bei hospitalisierten Kindern bezogen auf den Aufenthalt (Tage) auf der Intensivstation pro 100 Kinder und Tage unter künstlicher Beatmung pro 100 Kinder wurde durch die Prophylaxe mit Palivizumab nicht beeinflusst [20].

In einer weiteren placebokontrollierten Studie mit 1287 Kindern mit hämodynamisch signifikanten angeborenen Herzfehlern reduzierte eine monatliche Gabe über 5 Monate die Inzidenz der RSV-bedingten Krankenhausaufnahmen um 45 % (*p* = 0,003). Die RSV-Hospitalisierungsrate lag bei 9,7 % in der Placebogruppe und 5,3 % in der Palivizumab-Gruppe. Eine signifikante Reduzierung in der Palivizumab-Gruppe verglichen mit Placebo hinsichtlich der Gesamtzahl der Tage eines RSV-bedingten Krankenhausaufenthaltes (56 % Reduzierung, *p* = 0,003) und der Gesamtzahl der RSV-Tage mit einem erhöhten zusätzlichen Sauerstoffbedarf (73 % Reduzierung, *p* = 0,014) pro 100 Kinder konnte gezeigt werden [20].

Häufige Nebenwirkungen, die unter Palivizumab auftreten, sind Fieber, Hautausschlag und Reaktionen an der Injektionsstelle [20].

## Antikörpertherapien gegen das humane Immunschwächevirus (HIV)

Obwohl bei der Behandlung und Prävention von HIV-Infektionen mit antiretroviralen Medikamenten (ART) große Fortschritte erzielt wurden, hält die Epidemie mit etwa 38 Mio. Menschen, die aktuell mit HIV/Aids leben, und 1,7 Mio. Neuinfektionen pro Jahr an [21]. Eine passive Immunisierung durch die Gabe von Antikörpern könnte die therapeutischen Möglichkeiten erweitern. Diese Antikörper könnten entweder gegen zelluläre Komponenten, die beim Eintritt des HIV in die Zelle benötigt werden, oder gegen das Virus gerichtet sein. HIV-Infektionen zeigen aber eine sehr große Variabilität, die sich aus der hohen Mutationsrate des Virus ergibt. Allerdings stellen Antikörpertherapien eine letzte therapeutische Option für Patienten mit arzneimittelresistenten Viren dar. Genau für diese Indikation wurde der erste Antikörper für die Therapie von HIV-Infektionen im Oktober 2019 in der EU zugelassen. Dieser Antikörper, Ibalizumab, richtet sich nicht gegen das Virus, sondern bindet an den zellulären CD4-Rezeptor, blockiert ihn und hindert HIV am Eintritt in die CD4-Zelle. Ibalizumab ist nur für vorbehandelte Patienten zugelassen, deren Virus gegen andere HIV-Medikamente resistent ist. In einer Hauptstudie, an der 40 Erwachsene mit multiresistentem HIV teilnahmen, deren HIV-Behandlung fehlschlug, war die Viruslast (HIV-RNA-Kopien) im Blut bei 43 % der Patienten nach 25-wöchiger Kombination der Standardbehandlung mit Ibalizumab nicht nachweisbar (weniger als 50 HIV-RNA-Kopien/ml). Ähnliche Effekte wurden in einer zweiten Hauptstudie mit 113 Erwachsenen beobachtet, in der bei 44 % der Patienten, denen Erhaltungsdosen von Ibalizumab zur Standardbehandlung hinzugefügt wurden, nach 25 Wochen kein HIV nachweisbar war. Die häufigsten Nebenwirkungen von Ibalizumab (die bis zu 1 von 10 Patienten betreffen können) sind Hautausschlag, Durchfall, Schwindel, Kopfschmerzen, Übelkeit, Erbrechen und Müdigkeit [22].

## Antibakterielle monoklonale Antikörper

Antibakterielle MAB gegen eine Vielzahl von Bakterien können entweder gegen strukturelle Zelloberflächenkomponenten, wie z. B. Proteine oder Polysaccharide, oder bakterielle Exotoxine gerichtet sein. Während gegen Endotoxin gerichtete MAB einen Antigen-Antikörper-Komplex bilden, der primär durch das retikuloendotheliale System entfernt wird, ist die Pharmakodynamik der gegen strukturelle Zelloberflächenkomponenten gerichteten Antikörper abhängig von dem Antigen und dessen Rolle in der Pathogenese sowie der Struktur und des Isotyps des MAB. Monoklonale Antikörper, die gegen Oberflächenepitope gerichtet sind, zeigen entweder einen direkten bakteriziden Effekt oder erhöhen die bakterielle Clearance oder sie zeigen eine vom Immunsystem abhängige Zytotoxizität (Antikörper- oder Komplementabhängigkeit; [12, 23]).

In der EU steht seit 2017 ein MAB zur Vorbeugung von Rezidiven einer *Clostridium-difficile*-Infektion (CDI; EMA/CHMP: Zinplava-EPAR Summary for the Public (EMA/201086/2017), 2013) zur Verfügung. Bezlotoxumab ist ein humaner MAB, der mit hoher Affinität an *Clostridium-difficile*-Toxin‑B bindet und dessen Aktivität neutralisiert. Das Epitop des Toxin B, an das Bezlotoxumab bindet, ist hoch konserviert über die bekannten *Clostridium-difficile*-Stämme. Bezlotoxumab wird eingesetzt zur Verhinderung einer Rekurrenz bei CDI in Erwachsenen, die dafür ein erhöhtes Risiko aufweisen. Die Rekurrenz einer CDI wird mittels passiver Immunität gegen Toxine, die nach Auskeimen persistierender oder neu erworbener *Clostridium-difficile*-Sporen gebildet werden, verhindert. Die Wirksamkeit von Bezlotoxumab wurde in 2 randomisierten, doppelblinden, placebokontrollierten, multizentrischen Phase-3-Studien untersucht. Patienten wurden entweder mit Bezlotoxumab oder mit Placebo behandelt. Zusätzlich erhielten alle Patienten gleichzeitig eine 10- bis 14-tägige orale antibakterielle Therapie gegen die CDI. Vor Beendigung der antibakteriellen Therapie wurde eine Einmalinfusion Bezlotoxumab oder Placebo verabreicht und die Patienten wurden über einen Zeitraum von 12 Wochen nach Infusion nachbeobachtet. Die Rekurrenzrate konnte in der behandelten Gruppe statistisch signifikant gesenkt werden; besonders haben Patienten profitiert, bei denen Risikofaktoren für ein erhöhtes Risiko einer CDI vorlagen.

Das Sicherheitsprofil von Bezlotoxumab zeigt eine gute Verträglichkeit. Die häufigsten Nebenwirkungen, berichtet innerhalb der ersten 4 Wochen nach Infusion bei ≥4 % der Patienten, die mit Bezlotoxumab behandelt wurden, waren Übelkeit, Diarrhö, Fieber und Kopfschmerzen. Diese Nebenwirkungen wurden bei Patienten, die mit Placebo behandelt wurden, mit ähnlicher Häufigkeit berichtet [24].

## Monokloanale Antikörper zur symptomatischen Behandlung von Infektionskrankheiten

Zu Beginn der SARS-CoV-2-Pandemie stand eine kausale antivirale Therapie nicht zur Verfügung. Um die Folgen der Infektion, wie z. B. akutes Lungenversagen oder eine überschießende Immunreaktion, zu therapieren, wurden zahlreiche experimentelle Therapeutika eingesetzt. Unter diesen Therapeutika waren auch zahlreiche, insbesondere in immunologischen Indikationen zugelassene MAB, die als Interleukin-(IL-)Antagonisten zusammengefasst werden können. So geht die durch SARS-Coronaviren verursachte, akute lebensbedrohliche pulmonale Symptomatik mit erhöhten Werten von Interleukin-(IL-)2, IL‑6, IL‑7, IL‑1, granulozyten-makrophagen-kolonie-stimulierendem Faktor (GM-CSF), Tumornekrose-Faktor‑α, IFNγ IP10 (Interferon‑γ Inducible Protein 10), MCP‑1 (Monocyte Chemoattractant Protein 1) und MIP-1α- (Macrophage Inflammatory Protein 1α) einher [25].

Fallbeschreibungen von Patienten mit Pneumonien, die durch SARS-CoV oder MERS-CoV hervorgerufen wurden, zeigten eine Korrelation hoher Level proinflammatorischer Zytokine, wie z. B. IL‑6, und der radiologischen Schwere der Pneumonie [26, 27].

### IL-6-Rezeptorantagonisten

Der MAB Tocilizumab ist gegen den IL-6-Rezeptor gerichtet und wird unter anderem zur Behandlung verschiedener rheumatischer Erkrankungen sowie des Zytokin-Release-Syndroms (CRS) eingesetzt. Die Wirksamkeit von Tocilizumab zur Behandlung eines CRS wurde in einer retrospektiven Analyse klinischer Daten von Studien zu CAR-T-Zelltherapien zur Behandlung hämatologischer Malignitäten bewertet [28]. Erste Ergebnisse aus einer in China durchgeführten retrospektiven Fallsammlung zeigen bei 90 % der Patienten eine bildmorphologische Besserung unter Behandlung mit Tocilizumab, bei 15/20 Patienten kam es zu einer Verbesserung der respiratorischen Situation und weiterer Symptome [16]. Aufgrund des Wirkmechanismus des Antikörpers sind Infektionen ein bekanntes Risiko der Behandlung. Es wurden häufig vorübergehende oder intermittierende, leichte und mäßige Erhöhungen der Lebertransaminasen beobachtet (bei COVID-19 kann es zu einer Leberbeteiligung kommen, die evtl. durch Tocilizumab noch verschlechtert werden könnte; [28]). Ein Fallbericht von Hypertriglyzeridämie bei 2 mit Tocilizumab behandelten COVID-19-Patienten wurde veröffentlicht, bei einem der beiden Patienten kam es infolge der Behandlung zu einer Pankreatitis [29]. In einer prospektiven Pilotstudie mit 63 Patienten mit schwerer COVID-19 war die Gabe von Tocilizumab mit einer gesteigerten Überlebenswahrscheinlichkeit assoziiert (Hazard Ratio 2,2, 95 %-KI 1,3–6,7, *p* < 0,05). IL-6-Spiegel erwiesen sich in dieser Studie jedoch nicht als Prädiktorwert für die Mortalität, wohl aber die D‑Dimere bei Studienbeginn [30]. Zum jetzigen Zeitpunkt ist noch keine Evidenz für die Wirksamkeit von Tocilizumab bei COVID-19-Patienten vorgelegt worden [31, 32].

Sarilumab ist ein weiterer gegen den Interleukin-6-Rezeptor gerichteter MAB. Der 2017 von der EMA zugelassene Antikörper wurde für die Behandlung der rheumatoiden Arthritis entwickelt [33, 34]. Im Vergleich zu Tocilizumab (s. unten) weist Sarilumab eine längere Halbwertzeit auf; die Abstände zwischen den subkutanen Applikationen bei rheumatoider Arthritis betragen 14 statt 7 Tage. Sarilumab erhöht wie andere IL-6-Rezeptorantagonisten die Anfälligkeit für Infektionen; häufige Nebenwirkungen sind Leuko- und Thrombopenien. Der monoklonale Antikörper wird im Rahmen mehrerer klinischer COVID-19-Studien hinsichtlich seiner Wirksamkeit und Sicherheit untersucht. Eine multizentrische klinische Studie ist in Deutschland initiiert [35].

Siltuximab ist ein humanmuriner chimärer monoklonaler Antikörper, der humanes IL‑6 bindet und 2014 von der EMA zugelassen wurde [36]. Er wird zur Therapie des Morbus Castleman, einer seltenen lymphoproliferativen Erkrankung eingesetzt [37]. Eine Kontraindikation für die Behandlung mit Siltuximab stellt eine Infektion mit HIV oder humanem Herpesvirus 8 (HHV-8) dar. In mehreren klinischen Studien sollen die Wirksamkeit und Sicherheit von Siltuximab mittels intravenöser Einmalgabe bei Patienten mit COVID-19 und Hyperzytokinämie untersucht werden [25, 38]. In Spanien erhalten COVID-19-Patienten mit Pneumonie in einer randomisierten klinischen Studie der Phase 2 „open label“ entweder Siltuximab oder Methylprednisolon (nicht placebokontrolliert, 100 Teilnehmer, EudraCT Number: 2020-001413-20). In Belgien werden in einer 6‑armigen Studie mehrere IL-1- oder IL-6-Antagonisten, darunter auch Siltuximab, in einer klinischen Studie untersucht; Verbesserung der Oxygenierung und klinisches Outcome der Patienten sind Endpunkte der Studie (multizentrisch, randomisiert, offen, nicht placebokontrolliert, 342 Teilnehmer, EudraCT Number 2020-001500-41).

### IL-1-Antagonisten

Canakinumab ist ein humaner monoklonaler Antikörper gegen Interleukin-1β (IL-1β), der mit hoher Affinität eine Bindung des Interleukins an seinen Rezeptor unterbindet. Hierdurch wirkt die Substanz entzündungshemmend. Die Zulassung des Antikörpers besteht u. a. für folgende Autoimmunkrankheiten: Morbus Still, familiäres Mittelmeerfieber, Gichtarthritis [39]. Die Applikation erfolgt subkutan. Häufige Nebenwirkungen sind u. a. Atemwegsinfektionen [40]. In mehreren Ländern Europas und in den USA läuft eine klinische, vom Hersteller gesponsorte Studie zur Prüfung der Wirksamkeit und Sicherheit von Canakinumab bei COVID-19-Patienten (multizentrisch, RCT, Phase 3, CAN-COVID bzw. ClinicalTrials.gov NCT04362813, insgesamt 450 Teilnehmer). Eine weitere klinische Studie der Phase 2 in den USA untersucht, ob Canakinumab progressives Herz- und Lungenversagen bei COVID-19-Patienten verhindert. Die Patienten erhalten 300 mg oder 600 mg des monoklonalen Antikörpers intravenös (i. v.) oder ein Placebo (RCT, ClinicalTrials.gov NCT04365153, 45 Teilnehmer). In Italien erfolgt eine retrospektive und prospektive Beobachtungsstudie zu Canakinumab; die Patienten erhalten bzw. erhielten 150 mg subkutan (Nicht-RCT, 100 Teilnehmer, ClinicalTrials.gov NCT04348448).

Clazakizumab ist ein humanisierter monoklonaler Antikörper, der gegen Interleukin‑6 gerichtet ist und im Rahmen von klinischen Studien bei Patienten mit psoriatischer Arthritis und rheumatoider Arthritis untersucht wurde und bisher nicht zugelassen ist [41]. Ursprünglich war die Anwendung bei Patienten mit Graft-versus-Host-Disease geplant; der Antikörper soll eine 3‑ bis 120-mal stärkere Wirkung als Tocilizumab aufweisen [42]. Die Applikation erfolgt als Einmalgabe subkutan. Drei US-amerikanische RCT-Studien untersuchen Clazakizumab bei lebensbedrohlich erkrankten COVID-19-Patienten in den Dosierungen 25 mg oder 12,5 mg (ClinicalTrials.gov NCT04343989, Phase 2, RCT, 90 Teilnehmer) bzw. nur 25 mg (ClinicalTrials.gov NCT04381052: Phase 2/3, RCT, 30 Teilnehmer, ClinicalTrials.gov NCT04363502: Phase 2, RCT, 30 Teilnehmer). Eine weitere klinische Studie appliziert den monoklonalen Antikörper solchen Patienten, die noch keine invasive Beatmung benötigen, intravenös (ClinicalTrials.gov NCT04348500, Phase 2, RCT, 60 Teilnehmer). In Österreich können COVID-19-Patienten mit erhöhten Entzündungsbiomarkern und respiratorischer Verschlechterung im Rahmen der ACOVACT-Studie Clazakizumab erhalten (3 Untergruppen, ein Arm davon mit Clazakizumab, multizentrische Phase-2–3-Studie, randomisiert, unverblindet, gesamte geplante Teilnehmerzahl 500).

### Interferon-Gamma-Antagonisten

Emapalumab ist ein Anti-Interferon-Gamma-Antikörper (Anti-IFN-γ), der von der US-amerikanischen Arzneimittelbehörde Food and Drug Administration (FDA) die Zulassung zur Behandlung der primären hämophagozytären Lymphohistiozytose (HLH) erhalten hat [43]. Emapalumab bindet an lösliches und rezeptorgebundenes IFN‑γ, hierdurch wird die nachgeschaltete intrazelluläre Zytokinfreisetzung unterbunden. Häufige Nebenwirkungen sind eine erhöhte Infektionsneigung, Blutdruckerhöhungen, Hypokaliämie, gastrointestinale Nebenwirkungen, Exantheme und Fieber. Zur Behandlung von COVID-19-Patienten mit hyperinflammatorischer Zytokinausschüttung sollen die Wirksamkeit und Sicherheit von Emapalumab in Italien untersucht werden (multizentrische, 3‑armige, randomisierte, nichtplacebokontrollierte, „open label“ klinische Studie der Phase 2/3, ClinicalTrials.gov NCT04324021, Einschluss von 54 Patienten).

### Granulozyten-Monozyten-Kolonie-stimulierender-Faktor-(GM-CSF-)Antagonisten

Gimsilumab ist ein humaner monoklonaler Antikörper gegen den granulozyten-monozyten-kolonie-stimulierenden Faktor (GM-CSF), der im Rahmen von 2 klinischen Studien für den Einsatz bei Patienten mit Autoimmunerkrankungen mit Gelenkbeteiligung untersucht wurde. In einer klinischen Studie in den USA wird Gimsilumab bei COVID-19-Patienten mit akutem Atemnotsyndrom (ARDS) evaluiert werden; die multizentrische Studie ist randomisiert, doppelblind und placebokontrolliert angelegt; die Gabe des Antikörpers erfolgt intravenös (bisher in Studien subkutane Gaben). Es sollen 270 Patienten eingeschlossen werden (ClinicalTrials.gov NCT04351243).

Namilumab ist wie Gimsilumab ein humaner, monoklonaler Antikörper gegen GM-CSF, der in klinischen Studien bis Phase 3 für die Behandlung der rheumatoiden Arthritis und der Spondylitis ankylosans untersucht wird [44, 45]. Durch Blockade des proinflammatorischen GM-CSF wird die Zytokinausschüttung im Körper gehemmt, daher wird eine Wirkung bei COVID-19-Patienten vermutet [46]. In Italien wird Namilumab in einem Heilversuch eingesetzt; COVID-19-Patienten erhalten Namilumab bei rascher Verschlechterung des klinischen Zustands [47].

### Komplementinhibitoren

Akutes Lungenversagen wurde als Leitsymptom von SARS-CoV im Jahr 2012 bei dem Auftreten des verwandten SARS-CoV-2-Coronavirus und des Coronavirus mit respiratorischem Syndrom im Nahen Osten (MERS-CoV) beobachtet. Die Komplementaktivierung und die Komplementkomponente 5a (C5a), das proinflammatorische Anaphylatoxin sind an mehreren Punkten bei der Entwicklung einer durch pathogene Viren induzierten akuten Lungenerkrankung beteiligt [48]. Neue Erkenntnisse deuten darauf hin, dass die Aktivierung des Komplementsystems an der Pathogenese von coronavirus-(CoV-)bezogenem ARDS beteiligt ist und die Inhibition von C5 ein vielversprechender Ansatz bei SARS-CoV-2-vermittelten Erkrankungen sein kann [49].

Eculizumab ist zur Behandlung bei paroxysmaler nächtlicher Hämoglobinurie sowie atypischem hämolytisch-urämischem Syndrom (aHUS) zugelassen. Eculizumab hemmt die terminale Aktivierung des Komplementsystems. Die Grundlage des experimentellen Einsatzes bei mit SARS-CoV‑2 infizierten Patienten ist die Überlegung, dass die Immunantwort des Patienten eher als das Virus selbst zu ARDS und letztlich zum Tod führen kann [49]. Aufgrund seines Wirkmechanismus erhöht sich die Anfälligkeit des Patienten für eine Meningokokkeninfektion. Zur Verringerung des Infektionsrisikos müssten die Patienten mindestens 2 Wochen vor der Verabreichung von Eculizumab geimpft werden, es sei denn, das Risiko, das mit der Verzögerung der Therapie verbunden wäre, wiegt schwerer als die Risiken einer Meningokokkeninfektion. Patienten, deren Behandlung mit Eculizumab früher als 2 Wochen nach einer Meningokokkenimpfung beginnt, müssen bis 2 Wochen nach der Impfung eine geeignete Antibiotikaprophylaxe erhalten. Andere systemische Infektionen: Aufgrund seines Wirkungsmechanismus sollte die Therapie mit Eculizumab bei Patienten mit aktiven systemischen Infektionen mit Vorsicht durchgeführt werden. Patienten könnten eine erhöhte Anfälligkeit gegenüber Infektionen, insbesondere mit *Neisseria* und bekapselten Bakterien, aufweisen [50]. Der Hersteller führt eine Expanded-Access-Studie mit dem MAB bei COVID-19-Patienten durch: ClinicalTrials.gov NCT04355494. Auch eine weitere Firma hat eine Expanded-Access-Studie aufgelegt: ClinicalTrials.gov NCT04288713. In einem Arm der Studie CORIMUNO-19 sollen die Wirksamkeit und Sicherheit von Eculizumab bei COVID-19 untersucht werden (ClinicalTrials.gov NCT04346797, randomisierte, nichtplacebokontrollierte, „open label“ klinische Studie der Phase 2, 120 Teilnehmer).

Ravulizumab wurde zur Behandlung der paroxysmalen nächtlichen Hämoglobinurie und des atypischen hämolytisch-urämischen Syndroms entwickelt [51]. Der humanisierte monoklonale IgG2/4K-Antikörper bindet C5 der Komplementkaskade und verhindert dessen Aktivierung zu C5a, ein proinflammatorisches Anaphylatoxin [52, 53]. Die Applikation erfolgt gewichtsadaptiert und intravenös. Zwingend vor Behandlung empfohlen wird eine Impfung gegen Meningokokken, da bei Behandelten gehäuft Meningokokkeninfektionen auftraten. Ravulizumab wird in einer multizentrischen, offenen, randomisierten, kontrollierten Phase-3-Studie eingesetzt, um die Sicherheit und Wirksamkeit von Ravulizumab bei etwa 270 Patienten mit schwerer COVID-19, die aufgrund von Pneumonie, akuter Lungenschädigung (ALI) oder akutem Lungenversagen (ARDS) hospitalisiert sind, zu untersuchen (NCT04369469). Endpunkte sind das Überleben, die Notwendigkeit der mechanischen Beatmung, die Oxygenierung, die Dauer des Aufenthalts auf der Intensivstation. In der 3‑armigen Studie TACTIC‑R erhalten Patienten Ravulizumab, Barticinib oder beste supportive Therapie (ClinicalTrials.gov NCT04390464, Phase 4). Die Studie ist randomisiert, nicht placebokontrolliert, „open label“; es sollen 1167 Teilnehmer eingeschlossen werden.

IFX‑1 ist ein rekombinanter monoklonaler Antikörper gegen C5a des Komplementsystems. Das Komplementfragment C5a rekrutiert Granulozyten und Monozyten und erhöht die Gefäßpermeabilität. C5a löst gemeinsam mit anderen Fragmenten die lokale Inflammationsreaktion aus und fördert die Freisetzung von Histamin und Leukotrienen. Man geht davon aus, dass bei Viruspneumonien und auch anderen Lungenschädigungen die Aktivierung des Komplementsystems eine wesentliche Rolle bei der Entwicklung eines ARDS spielt [54]. Im Tiermodell mit vogelgrippe-(H7N9-)infizierten Affen konnten die histopathologischen Lungenschäden durch Gabe von Anti-C5a reduziert werden, ebenso sanken die Level der Entzündungsmarker und die Virustiter in den infizierten Lungen. Sun et al. sehen die Inhibition der Komplementkaskade als vielversprechende additive Therapieoption bei viralen Pneumonien [55]. Hohe Level von C5a und exzessive Aktivierung der Komplementkaskade sehen Wang et al. als wesentliche Auslöser des ARDS bei viralen Lungenentzündungen, wie beispielsweise SARS. C5a habe eine Schlüsselstellung innerhalb des Komplementsystems, daher sei die Blockade von C5a bei virusinduziertem ARDS eine therapeutische Option [48]. In den Niederlanden werden derzeit Patienten mit schwerer COVID-19 für eine klinische Phase-II/III-Studie mit IF‑X rekrutiert. Angaben zur Dosierung sind nicht öffentlich zugänglich. Die Studie ist multizentrisch, randomisiert, „open label“ und schließt 130 Teilnehmer ein (ClinicalTrials.gov NCT04333420).

## Ausblick

Breit neutralisierende Antikörper (bnAB), die gegen die Mehrheit der derzeit weltweit im Umlauf befindlichen HIV-Isolate wirksam sind, haben das höchste Potenzial, therapeutisch aktiv zu sein. Die Antikörper können verschiedene funktionelle Eigenschaften haben, wie z. B. die Neutralisierung freier Viren, die Eliminierung infizierter Zellen und die Hemmung der Zell-zu-Zell-Übertragung von HIV‑1 [56, 57]. Aufgrund ihrer Produkteigenschaften, wie einer langen Halbwertszeit, einer ausgezeichneten Sicherheit und der Einbindung der Immunantwort des Wirts, könnten Antikörper die therapeutischen Möglichkeiten erweitern. Der Schutz durch bnAB ist aber nur transient und müsste im Falle der HIV-Prävention periodisch wiederholt werden. Es können ähnlich wie bei ART unter Antikörpertherapie auch antikörperresistente Viren entstehen. Eine Kombinationstherapie mit mehreren bnAB würde auch hier Abhilfe schaffen.

Die Entwicklung von bnAB zur Behandlung von HIV-Infektionen kann als Grundlage der Entwicklung von MAB gegen unterschiedliche Viruserkrankungen dienen. Insbesondere ist hier die Entwicklung von MAB zur Behandlung von Infektionen mit dem Ebolavirus, Influenzaviren oder Coronaviren (SARS-CoV, MERS und SARS-CoV-2) zu nennen [58–61].

Auch Toxine sind ausgezeichnete Zielantigene für Antikörper, da sie sich normalerweise strukturell von den Selbstantigenen unterscheiden, die von den Wirtszellen exprimiert werden. Ein spezifischer Antikörper kann die einzige Substanz sein, die ein bestimmtes Toxin neutralisieren kann, da antimikrobielle Arzneimittel zwar die Mikroben abtöten, die zirkulierenden Toxine jedoch nicht eliminieren können. Antikörper sind daher auch in diesem Anwendungsbereich attraktiv für die therapeutische Entwicklung [62]. Die Entwicklung von MAB gegen Toxine wird vor allem von den USA dominiert, um beispielsweise bei bioterroristischen Angriffen eine Therapie oder sekundäre Prophylaxe vorrätig zu haben [45].
